# Axillary Web Syndrome Beyond Breast Cancer: Expanding the Clinical Spectrum Through Illustrative Cases and Contemporary Evidence

**DOI:** 10.3390/jcm15176755

**Published:** 2026-08-31

**Authors:** Nathalia Cardoso Oliveira, José Marcelo Corassa, Fanilda Souto Barros, Brenno Augusto Seabra de Mello Netto, Anke Bergmann, Joana Storino

**Affiliations:** 1 Faculdade Israelita de Ciências da Saúde Albert Einstein, Hospital Israelita Albert Einstein, São Paulo 05653-000, SP, Brazil; 2 Maravasc for Health Science, Av. Maestro João Nunes, 02, São Luís 65076-730, MA, Brazil; 3 Hospital Santa Paula, Vitória 29090-640, ES, Brazil; 4 Instituto Fanilda Barros, Vitória 29050-545, ES, Brazil; 5 Seabra Excelência Vascular, Vitória 29052-210, ES, Brazil; 6 Instituto Nacional de Câncer, Rio de Janeiro 20230-130, RJ, Brazil; 7 Hospital Mater Dei, Belo Horizonte 30190-09, MG, Brazil

**Keywords:** axillary web syndrome, lymphatic disorders, ultrasonography, differential diagnosis, upper extremity, breast cancer surgery

## Abstract

**Background/Objectives**: This study aimed to expand the recognized clinical spectrum of Axillary Web Syndrome (AWS) by integrating illustrative oncologic and non-oncologic cases with a contemporary review of the evidence regarding its epidemiology, diagnosis, pathophysiology, and management. **Methods**: This study combines a retrospective case series of three patients diagnosed with AWS with a contemporary narrative review of the literature. The series included one patient following breast cancer surgery and two patients without oncologic history who developed AWS after repetitive upper-limb activity. Clinical presentation, ultrasonographic findings, treatment, and outcomes were analyzed. The literature review contextualized current evidence on the epidemiology, pathophysiological mechanisms, diagnostic approach, imaging assessment, differential diagnosis, and therapeutic management of AWS. **Results**: Three patients with AWS were identified, including one classic postoperative breast cancer case and two non-oncologic presentations following repetitive upper-limb activity. All patients presented with palpable subcutaneous cords, pain, and restricted shoulder abduction. Ultrasonography demonstrated no evidence of superficial thrombophlebitis or other structural abnormalities, supporting the exclusion of alternative diagnoses rather than confirming AWS. Conservative treatment consisting of physiotherapy, stretching exercises, and progressive shoulder mobilization resulted in symptom resolution and functional recovery in all cases. The literature review demonstrated that AWS may occur in a broader range of oncologic and non-oncologic settings than traditionally recognized, supporting the hypothesis that localized lymphovascular injury may contribute to a shared pathophysiological pathway across different clinical settings. **Conclusions**: AWS should be considered in the differential diagnosis of patients presenting with painful axillary cords and shoulder dysfunction, regardless of a history of breast cancer. Early clinical recognition, appropriate use of ultrasonography to exclude competing diagnoses, and timely conservative rehabilitation are essential to optimize functional recovery. These findings support a broader conceptual framework in which AWS may represent a clinical syndrome of localized lymphovascular injury rather than a complication restricted exclusively to breast cancer surgery.

## 1. Introduction

Axillary Web Syndrome (AWS), also referred to as axillary cording or lymphatic cording, is an underrecognized clinical condition characterized by the development of palpable or visible subcutaneous cords extending from the axilla along the medial aspect of the upper limb. Clinically, patients typically present with pain, shoulder stiffness, and limitation of abduction, leading to functional impairment that may significantly affect daily activities and postoperative recovery [1,2,3].

Since its original description in 2001, AWS has been regarded primarily as a complication of breast cancer surgery, particularly following axillary lymph node dissection, although it has also been reported after sentinel lymph node biopsy [1,4]. Reported incidence ranges from approximately 6% to more than 70%, reflecting differences in study design, timing of postoperative assessment, examiner expertise, and the absence of standardized diagnostic criteria [5,6,7,8]. Despite its generally favorable prognosis, AWS may delay rehabilitation, prolong pain, and compromise shoulder function when not promptly recognized and managed.

Regional data also highlight the influence of systematic clinical assessment on AWS detection. In a Brazilian prospective cohort of 173 women undergoing breast cancer surgery, Figueira et al. reported a cumulative incidence of 90.9% within 180 days, with 66.1% of cords identified by the seventh postoperative day. More than 70% of the cords were palpable, and pain was reported in 39.7% of patients. Lymphadenectomy and hypertension were associated with an increased risk of AWS, whereas the observed association between diabetes and a lower risk of cord development requires further investigation [9].

Although the clinical presentation of AWS has been recognized for more than two decades, its pathophysiology remains incompletely understood. Early anatomical and histopathological investigations suggested that the characteristic cords originated from injured superficial lymphatic vessels undergoing thrombosis and fibrosis [10,11,12]. More recent studies, however, have demonstrated that vascular remodeling, inflammatory changes, connective tissue fibrosis, and arterial alterations may also contribute to cord formation, supporting the concept that AWS represents a multifactorial lymphovascular disorder rather than an exclusively lymphatic condition [13,14,15].

An equally important advance has been the recognition that AWS is not restricted to breast cancer surgery. During the last decade, isolated reports have described the syndrome after melanoma surgery, venous blood sampling, dermatologic procedures, repetitive upper-limb activity, and even in patients without previous surgery or malignancy [16,17,18,19]. These observations challenge the traditional perception of AWS as an exclusively postoperative oncologic complication and suggest that localized lymphovascular injury, regardless of its etiology, may represent the common pathogenic mechanism. Consequently, physicians from multiple specialties—including vascular surgery, rehabilitation medicine, orthopedics, sports medicine, dermatology, and primary care—should be familiar with its presentation.

Despite increasing recognition of atypical presentations, AWS remains fundamentally a clinical diagnosis. Careful inspection and palpation of the axilla during shoulder abduction remain the cornerstone of diagnosis, whereas ultrasonography should primarily be regarded as a complementary tool to exclude competing diagnoses—particularly superficial thrombophlebitis—rather than as a confirmatory examination [2,20,21,22,23]. Limited awareness outside breast cancer care frequently results in delayed diagnosis, unnecessary investigations, and postponement of appropriate rehabilitation. Management is predominantly conservative, with physiotherapy, stretching exercises, progressive shoulder mobilization, and manual therapy representing the main therapeutic approaches [24,25]. Early recognition and rehabilitation may help minimize prolonged functional impairment; however, standardized preventive strategies for AWS have not yet been established.

Despite these advances, the literature remains predominantly focused on postoperative breast cancer populations, and the clinical spectrum, underlying mechanisms, and diagnostic implications of AWS in non-oncologic settings remain insufficiently characterized. The present study aims to broaden the recognized clinical spectrum of Axillary Web Syndrome by combining illustrative cases from oncologic and non-oncologic settings with a contemporary review of the current evidence regarding its epidemiology, pathophysiology, diagnosis, imaging assessment, differential diagnosis, and management. We hypothesize that AWS may represent a clinical syndrome of localized lymphovascular injury rather than a complication restricted to breast cancer surgery. This evolving concept forms the basis of the present review.

## 2. Materials and Methods

### 2.1. Study Design

This study combines a retrospective case series with a contemporary narrative review of the literature to illustrate the expanding clinical spectrum of Axillary Web Syndrome (AWS). All patients diagnosed with AWS and included in this retrospective case series had been evaluated during routine clinical care. Diagnosis was established clinically based on medical history and physical examination, characterized by the presence of palpable or visible subcutaneous cords extending from the axilla, accompanied by pain and limitation of shoulder movement. Clinical assessments and diagnoses were performed by experienced physicians (J.M.C. and B.A.S.M.N.) based on medical history and physical examination. No standardized pain scale, goniometric measurement of shoulder range of motion, or validated functional assessment instrument was systematically applied during the original clinical evaluations. Ultrasonography was performed in all patients using a Philips EPIQ Elite ultrasound system with a high-frequency linear transducer (12 MHz) (Philips Ultrasound, Inc., Bothell, WA, USA) to exclude alternative diagnoses, particularly superficial thrombophlebitis and other structural abnormalities, rather than to confirm the diagnosis of AWS. Clinical and functional recovery during follow-up was defined clinically by resolution of pain or discomfort and restoration of unrestricted shoulder movement, as documented during routine clinical reassessment.

### 2.2. Case Selection

Patients presenting with clinical findings consistent with AWS were eligible regardless of the underlying etiology. All patients diagnosed with AWS at the center between January 2024 and December 2025 were included; no sampling or intentional selection of cases was performed. Research data were retrospectively collected from the medical records in 2025, after ethical approval. Clinical photographs had been obtained during routine clinical care for medical-record documentation and follow-up and were subsequently included in the retrospective analysis.

Inclusion criteria were a clinical diagnosis of AWS characterized by a palpable or visible subcutaneous cord extending from the axilla along the medial aspect of the upper limb, associated with pain and/or restriction of shoulder movement, and sufficient clinical documentation available for retrospective evaluation. Patients were excluded if the available clinical information was insufficient to establish the diagnosis of AWS or if an alternative diagnosis better explained the axillary or upper-limb findings. Clinical information was obtained through review of medical records, imaging examinations, and standardized photographic documentation collected during routine clinical practice.

Demographic and clinical variables included age, sex, relevant medical and surgical history, clinical context preceding AWS onset, presenting symptoms, physical examination findings, ultrasonographic findings, treatment, and clinical outcome. No specific laboratory testing was required for the diagnosis of AWS, which was established clinically.

### 2.3. Literature Review

A contemporary narrative review was performed using PubMed/MEDLINE to identify publications addressing the epidemiology, pathophysiology, diagnosis, imaging findings, differential diagnosis, and treatment of AWS. The literature search was last updated on 29 June 2026 and included combinations of the terms “axillary web syndrome”, “axillary cording”, “lymphatic cording”, “breast cancer”, “breast surgery”, “non-oncologic”, “ultrasonography”, “pathophysiology”, “diagnosis”, “rehabilitation”, and “treatment”. Priority was given to systematic reviews, prospective studies, and landmark publications considered relevant to the objectives of this manuscript.

Relevant publications were identified through a review of peer-reviewed literature, prioritizing original investigations, systematic reviews, randomized controlled trials, cohort studies, case series, and illustrative case reports addressing atypical clinical presentations, advances in imaging, pathophysiological mechanisms, and rehabilitation strategies.

Because the primary objective of this study was to provide an updated clinical overview integrated with illustrative cases, the review was conducted as a narrative synthesis rather than as a systematic review. Consequently, no formal systematic review methodology, predefined search strategy, or meta-analytic approach was applied. Instead, the available evidence was critically appraised to support interpretation of the presented cases and discussion of current concepts regarding AWS.

The literature review was intended to support the clinical interpretation of the presented cases rather than to estimate pooled effects or provide quantitative evidence synthesis. Accordingly, this manuscript should be interpreted as a contemporary clinical review supported by illustrative cases rather than as a systematic review.

### 2.4. Data Analysis

Given the descriptive nature and small sample size of this case series, no inferential statistical analyses were performed. Demographic, clinical, imaging, treatment, and outcome data were summarized descriptively. Accordingly, statistical hypothesis testing, *p*-values, and dedicated statistical software were not applicable.

### 2.5. Ethical Considerations

The study was approved by the Research Ethics Committee of CIAS—UNIMED Vitória, Vitória, ES, Brazil, on 25 March 2025 (approval No. 7.462.937) and conducted in accordance with the ethical principles of the Declaration of Helsinki. Retrospective research data collection from the medical records was performed only after ethical approval had been obtained. Written informed consent for publication of anonymized clinical information and accompanying clinical photographs was obtained from all participants prior to publication.

During manuscript preparation, OpenAI’s ChatGPT (GPT-5.6 Sol; OpenAI, San Francisco, CA, USA) was used solely to assist with English-language editing, text organization, and improvement of linguistic fluency. All scientific content, interpretation, critical revision, and final approval of the manuscript were performed by the authors.

## 3. Results

### 3.1. Overview

Three patients diagnosed with AWS were included in this case series. One patient developed AWS following breast cancer surgery, while two presented with non-oncologic manifestations, including one case associated with repetitive upper-limb physical activity. All patients presented with palpable subcutaneous cords extending from the axilla and varying degrees of pain and restricted shoulder movement. Ultrasonography was performed in all cases and showed no evidence of superficial thrombophlebitis or other structural abnormalities, supporting the exclusion of alternative diagnoses.

**Figure 1:**
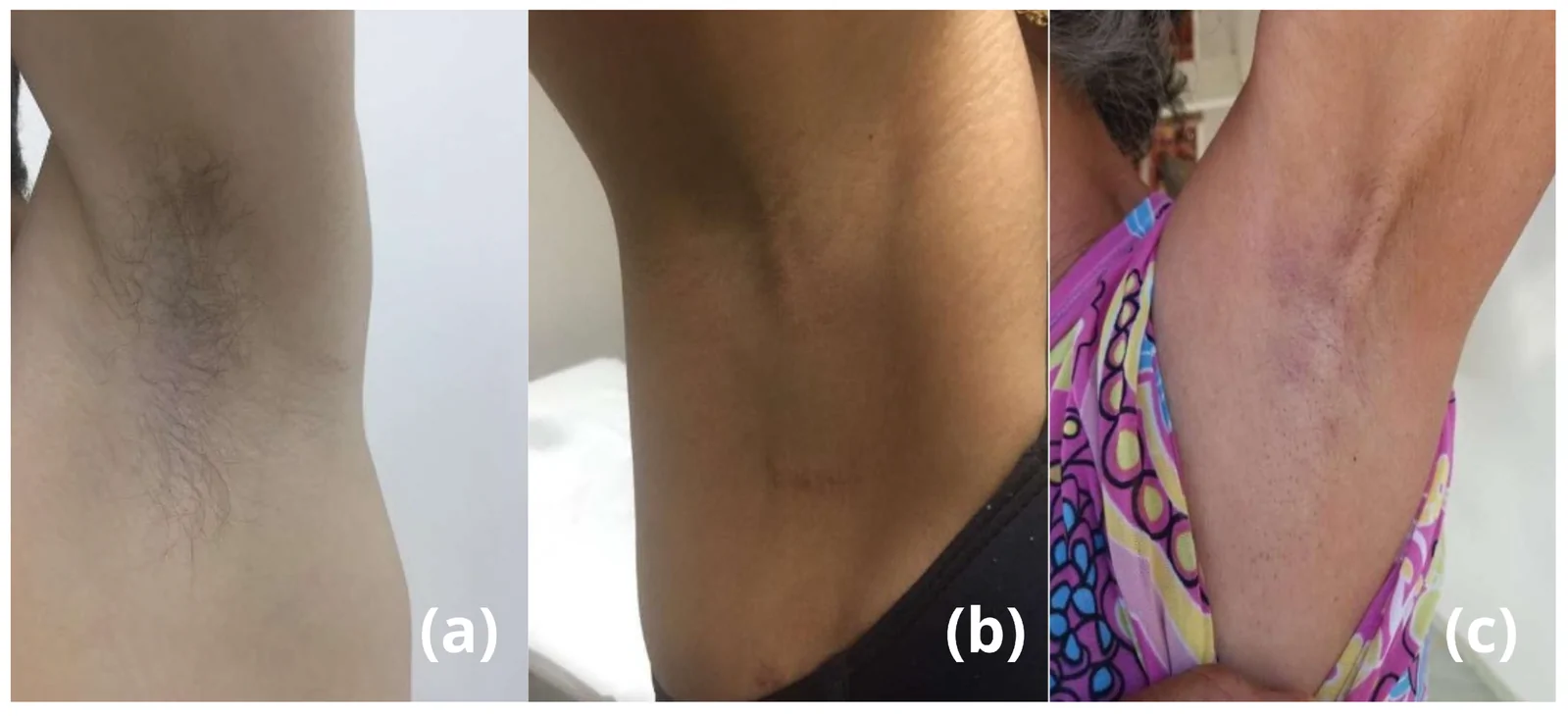
Representative clinical manifestations of AWS: (**a**) AWS in a male patient following intense squash activity. (**b**) Classical postoperative presentation following breast cancer surgery. (**c**) Non-oncologic presentation associated with repetitive occupational upper-limb activity. These representative cases illustrate the clinical spectrum of AWS across oncologic and non-oncologic settings.

The main demographic characteristics, clinical findings, imaging results, management strategies, and outcomes are summarized in Table 1. Representative clinical manifestations of the three cases are shown in Figure 1.

### 3.2. Case 1

A previously healthy male patient presented with acute pain and restricted left shoulder abduction immediately after participating in an extended squash tournament. He denied previous trauma, surgical procedures, systemic disease, or recent infection.

Physical examination revealed a palpable and visible subcutaneous cord extending from the left axilla along the medial aspect of the upper arm, becoming more evident during shoulder abduction (Figure 1a). Shoulder elevation reproduced the patient’s symptoms and demonstrated functional limitation.

Given the unusual presentation in an otherwise healthy individual without previous axillary surgery, the initial differential diagnoses included superficial thrombophlebitis, muscle strain, lymphangitis, and soft tissue injury.

Ultrasonography demonstrated no evidence of superficial thrombophlebitis or other structural abnormalities, supporting exclusion of alternative diagnoses rather than confirmation of AWS.

The diagnosis was established clinically, and conservative management consisting of physiotherapy, stretching exercises, myofascial release, and progressive shoulder mobilization was initiated. The patient experienced progressive symptom improvement, with complete clinical and functional recovery within two weeks of follow-up.

### 3.3. Case 2

A woman undergoing treatment for breast cancer developed progressive pain and limitation of shoulder movement approximately six weeks after mastectomy. Physical examination demonstrated a palpable cord extending from the ipsilateral axilla toward the medial aspect of the upper arm, consistent with AWS (Figure 1b).

Considering the broad differential diagnosis of postoperative shoulder pain after breast cancer surgery, superficial thrombophlebitis, postoperative fibrosis, lymphatic complications, and local inflammatory conditions were initially considered.

Ultrasonography demonstrated no evidence of superficial thrombophlebitis or other structural abnormalities, supporting exclusion of alternative diagnoses.

The diagnosis of AWS was established clinically, and conservative rehabilitation with manual therapy, myofascial release, stretching exercises, and progressive shoulder mobilization was initiated. Progressive improvement in pain and shoulder mobility was observed, with clinical and functional recovery within two weeks of follow-up.

### 3.4. Case 3

A woman without previous oncologic disease presented with a palpable axillary cord after an exceptionally strenuous workday involving prolonged and repetitive upper-limb activity in a commercial kitchen. She denied previous surgery, trauma, or recent infection.

Clinical examination demonstrated a visible cord extending from the axilla along the medial aspect of the arm, associated with mild discomfort and limitation of shoulder movement (Figure 1c).

Because the presentation occurred in the absence of breast surgery, the initial differential diagnoses included superficial thrombophlebitis, muscle injury, lymphangitis, and axillary lymphadenopathy.

Ultrasonography demonstrated no evidence of superficial thrombophlebitis or other structural abnormalities, supporting exclusion of alternative diagnoses rather than confirmation of AWS. Mammography was also performed and showed no suspicious breast abnormalities.

A clinical diagnosis of AWS was established, and conservative treatment with physiotherapy, stretching exercises, and progressive shoulder mobilization was recommended. Symptoms progressively resolved, with complete clinical and functional recovery within two weeks of follow-up.

### 3.5. Overall Findings

The three cases illustrate the heterogeneous clinical spectrum of AWS. One patient developed AWS following breast cancer surgery, while two presented with non-oncologic manifestations temporally associated with intense or repetitive upper-limb physical activity. In all cases, diagnosis was established clinically following exclusion of alternative conditions, particularly superficial thrombophlebitis, underscoring the importance of physician awareness beyond the traditional postoperative breast cancer context.

## 4. Discussion

### 4.1. Principal Findings

The present study expands the recognized clinical spectrum of AWS by illustrating that the condition is not exclusively associated with breast cancer surgery. By integrating three illustrative clinical cases with a contemporary review of the available evidence, our findings reinforce three central concepts. First, AWS may represent a clinical syndrome associated with localized lymphovascular injury arising in different clinical settings rather than a complication restricted to oncologic surgery. Second, diagnosis remains fundamentally clinical, with careful history taking and physical examination representing the cornerstone of evaluation, whereas ultrasonography should primarily be used to exclude alternative diagnoses rather than to confirm the syndrome itself. Third, increasing awareness of AWS among physicians from multiple specialties may facilitate earlier diagnosis, reduce unnecessary investigations, and promote timely conservative rehabilitation, ultimately improving functional recovery and patient outcomes.

Since its original description by Moskovitz et al. (2001) [1], the understanding of AWS has evolved substantially. Initially regarded as an uncommon and self-limiting postoperative complication following axillary lymph node dissection for breast cancer, subsequent anatomical, histopathological, imaging, and clinical investigations have demonstrated that the syndrome is considerably more frequent than originally recognized and biologically more complex than initially proposed [1,4,10,11,12,13,14,15]. Rather than representing an isolated lymphatic disorder, accumulating evidence increasingly supports AWS as a multifactorial process involving lymphatic injury, inflammation, fibrosis, connective tissue remodeling, and, in selected patients, vascular alterations. The present study further contributes to this evolving concept by demonstrating that similar clinical manifestations may occur following both oncologic and non-oncologic lymphovascular injury.

Taken together, our findings support a broader conceptual interpretation of AWS as a clinical phenotype of localized lymphovascular injury. This perspective not only explains the increasing number of atypical presentations reported in recent years but also highlights the importance of recognizing the syndrome beyond the traditional context of breast cancer surgery.

### 4.2. Epidemiology and Risk Factors

Although Axillary Web Syndrome (AWS) has traditionally been recognized as a postoperative complication of breast cancer surgery, its epidemiology remains incompletely defined. Reported incidence varies considerably across the literature, ranging from approximately 6% to more than 70%, largely reflecting differences in surgical procedures, timing of postoperative assessment, examiner expertise, study design, and the absence of universally accepted diagnostic criteria [5,7,8]. Importantly, studies incorporating systematic postoperative physical examination by trained clinicians consistently report higher incidence rates than those relying solely on spontaneous patient complaints, suggesting that AWS remains substantially underdiagnosed in routine clinical practice.

The syndrome occurs most frequently after axillary lymph node dissection but is also observed following sentinel lymph node biopsy, indicating that even limited disruption of superficial lymphatic pathways may be sufficient to trigger cord formation in susceptible individuals [1,4,7]. Most patients develop symptoms within the first weeks after surgery, although delayed presentations have also been described [5,8]. While AWS is generally considered a self-limiting condition, delayed diagnosis may prolong pain, restrict shoulder mobility, impair postoperative rehabilitation, and negatively affect quality of life [2,3].

Several factors have been associated with an increased risk of developing AWS, although the available evidence remains heterogeneous. More extensive axillary surgery, younger age, lower body mass index, greater postoperative shoulder dysfunction, and the presence of lymphedema have all been reported as potential risk factors [7,8,20,21,22,26,27,28]. Nevertheless, these associations have not been consistently reproduced across all studies, emphasizing the multifactorial nature of the syndrome and the methodological variability of the available evidence. The lack of standardized diagnostic criteria continues to limit direct comparison among epidemiological studies and probably contributes to underestimation of the true disease burden.

Another important epidemiological consideration is the progressive recognition of AWS outside the traditional breast cancer setting. Reports describing the syndrome after melanoma surgery, venipuncture, punch biopsy, repetitive upper-limb activity, and other forms of localized tissue injury suggest that the condition is likely underrecognized in non-oncologic populations [16,17,18,19].

### 4.3. Axillary Web Syndrome Beyond Breast Cancer

Although Axillary Web Syndrome (AWS) has historically been regarded as a postoperative complication of breast cancer surgery, accumulating evidence over the past decade has progressively expanded its recognized clinical spectrum. Increasing reports demonstrate that AWS should no longer be viewed as a condition exclusively associated with oncologic procedures but rather as a clinical syndrome resulting from localized lymphovascular injury, irrespective of the underlying etiology [4,11,12].

The first observations supporting this broader perspective emerged from studies involving patients undergoing surgical treatment for melanoma. Schuitevoerder et al. (2016) [16] demonstrated that AWS may occur after sentinel lymph node biopsy for melanoma, producing clinical manifestations indistinguishable from those observed after breast cancer surgery. These findings reinforced the concept that disruption of superficial lymphatic pathways, rather than the underlying malignancy itself, represents the fundamental event responsible for cord formation.

Subsequent reports have continued to broaden the spectrum of recognized triggering events. AWS has been described following routine venous blood sampling [17], punch biopsy [18], and, more recently, in pediatric patients without previous surgery or malignancy [19]. Collectively, these observations indicate that even minor localized injuries involving superficial lymphovascular structures may initiate a common inflammatory and reparative process culminating in the development of the characteristic palpable cords.

Mechanical overload has been proposed as a possible non-surgical context for AWS, although the available evidence remains limited to isolated reports [13,17,29]. Because a temporal association does not establish causality, repetitive upper-limb activity should be interpreted as a potential precipitating factor rather than a demonstrated cause. Repetitive mechanical stress could plausibly contribute to localized lymphovascular injury in susceptible individuals, but this hypothesis requires further investigation. Consequently, AWS is likely underrecognized outside oncology practice, where physicians may be less familiar with its characteristic clinical presentation.

The present case series further strengthens this evolving concept. Two of the three patients presented with AWS in the absence of breast cancer or previous axillary surgery. One patient presented immediately after prolonged squash practice, whereas another developed symptoms following an exceptionally strenuous workday involving prolonged repetitive upper-limb activity in a commercial kitchen. Although the temporal relationship in these two cases is clinically noteworthy, it does not establish a causal association between repetitive activity and AWS. In both cases, the diagnosis was established through the identification of the characteristic palpable axillary cord during physical examination, while duplex ultrasonography excluded superficial thrombophlebitis and other structural abnormalities. These observations closely parallel the growing body of literature describing AWS after melanoma surgery, venipuncture, punch biopsy, repetitive physical activity, and other non-classical clinical scenarios.

Recognition of AWS beyond the oncologic setting has important practical implications. Because the syndrome continues to be strongly associated with breast cancer surgery in everyday clinical practice, patients presenting in other contexts are at substantial risk of delayed diagnosis or misdiagnosis. Superficial thrombophlebitis, Mondor’s disease, lymphangitis, postoperative fibrosis, muscle strain, tendon injury, adhesive capsulitis, and axillary lymphadenopathy should all be considered during the diagnostic evaluation, particularly when inflammatory, vascular, musculoskeletal, or nodal abnormalities are suspected [2,23,30]. However, careful physical examination remains the key element distinguishing AWS from these alternative conditions.

Taken together, the available evidence suggests that AWS should no longer be interpreted solely as a postoperative complication of breast cancer surgery. Instead, it appears to represent a recognizable clinical phenotype of localized lymphovascular injury, characterized by palpable axillary cording, pain, and restricted shoulder mobility, regardless of the initiating mechanism. This broader conceptual framework not only explains the increasing number of atypical cases reported in recent years but also has important implications for diagnosis, epidemiological research, and multidisciplinary patient care.

Representative reports describing AWS outside the traditional breast cancer setting are summarized in Table 2.

### 4.4. Clinical Presentation, Differential Diagnosis, and Imaging Assessment

Axillary Web Syndrome (AWS) remains fundamentally a clinical diagnosis. Although imaging has substantially contributed to understanding its anatomical and pathophysiological basis, diagnosis continues to rely primarily on a careful clinical history and physical examination. Patients typically present with acute or progressive axillary pain, restricted shoulder range of motion, and one or more palpable or visible subcutaneous cords extending from the axilla along the medial aspect of the upper arm. These cords usually become more evident during shoulder abduction and, in more extensive cases, may extend to the forearm or even the base of the thumb [10,11,12,20,21,27,32,33].

Because no laboratory or imaging examination is pathognomonic for AWS, meticulous physical examination remains the cornerstone of diagnosis. Dynamic inspection during shoulder abduction is particularly important, as the cords may not be readily visible with the arm at rest. Likewise, systematic palpation of the axilla and medial arm is essential, since failure to actively search for the characteristic cord may result in delayed diagnosis or misinterpretation of symptoms, especially in patients without a previous history of breast cancer surgery [30,34].

The principal differential diagnosis is superficial thrombophlebitis, as both conditions may present with painful palpable cords. However, important clinical differences should be recognized. Superficial thrombophlebitis usually follows the anatomical course of a superficial vein and is frequently accompanied by localized erythema, tenderness, warmth, and ultrasonographic evidence of venous thrombosis. In contrast, AWS typically presents as a thin, tense subcutaneous cord that becomes more prominent during shoulder abduction and is not associated with inflammatory skin changes or venous thrombosis on duplex ultrasonography [1,3,28,34,35,36].

Additional differential diagnoses include Mondor’s disease, lymphangitis, postoperative fibrosis, muscle strain, tendon injury, adhesive capsulitis, and axillary lymphadenopathy. Although these conditions may mimic certain clinical features of AWS, they differ substantially in their underlying pathophysiology, therapeutic approach, and prognosis. Recognition of these distinctions is essential to avoid unnecessary investigations and inappropriate treatment [2,23,30].

The principal clinical and ultrasonographic features useful for distinguishing AWS from its major differential diagnoses are summarized in Table 3.

Although AWS is primarily diagnosed clinically, imaging plays an important complementary role by excluding competing diagnoses rather than confirming the syndrome itself. Among the available imaging modalities, ultrasonography remains the most accessible, noninvasive, and widely used examination in routine clinical practice, particularly when patients present with painful palpable cords raising suspicion for superficial thrombophlebitis or other vascular disorders [3,13,14,28,37,38,39,40,41].

Early ultrasonographic studies reported inconsistent visualization of the characteristic cords, largely because of their small caliber and variable anatomical composition. Depending on the stage of the disease and the tissues involved, the cords may appear as hypoechoic or hyperechoic linear structures within the superficial subcutaneous tissue, whereas many patients demonstrate no specific sonographic abnormality. Consequently, normal ultrasonographic findings should never be interpreted as excluding AWS [28,40,42,43].

Beyond conventional ultrasonography, lymphoscintigraphy and histopathological investigations have provided important insights into the biological mechanisms underlying AWS. Lymphoscintigraphic studies have demonstrated abnormalities in superficial lymphatic drainage, whereas histopathological analyses have identified lymphatic injury, fibrosis, inflammatory changes, and vascular remodeling, reinforcing the concept that AWS involves multiple tissue compartments rather than isolated lymphatic dysfunction [1,12,14,15,44]. Nevertheless, these techniques remain primarily research tools and have not altered the diagnostic approach in routine clinical practice.

The greatest clinical value of duplex ultrasonography lies in excluding clinically relevant differential diagnoses. Evaluation of the superficial venous system allows reliable exclusion of superficial thrombophlebitis, venous thrombosis, and other structural abnormalities when clinically indicated. This distinction is particularly important because superficial thrombophlebitis frequently requires specific therapeutic management, whereas AWS is treated conservatively.

The three patients included in the present series clearly illustrate this diagnostic strategy. In every case, the diagnosis of AWS was established clinically through the identification of the characteristic palpable cord during physical examination, while ultrasonography demonstrated no evidence of superficial thrombophlebitis or other structural abnormalities. These findings supported the exclusion of relevant competing diagnoses and the decision to pursue conservative management. Rather than excluding the diagnosis, these normal imaging findings increased diagnostic confidence by ruling out competing conditions and supporting conservative management.

**Figure 2:**
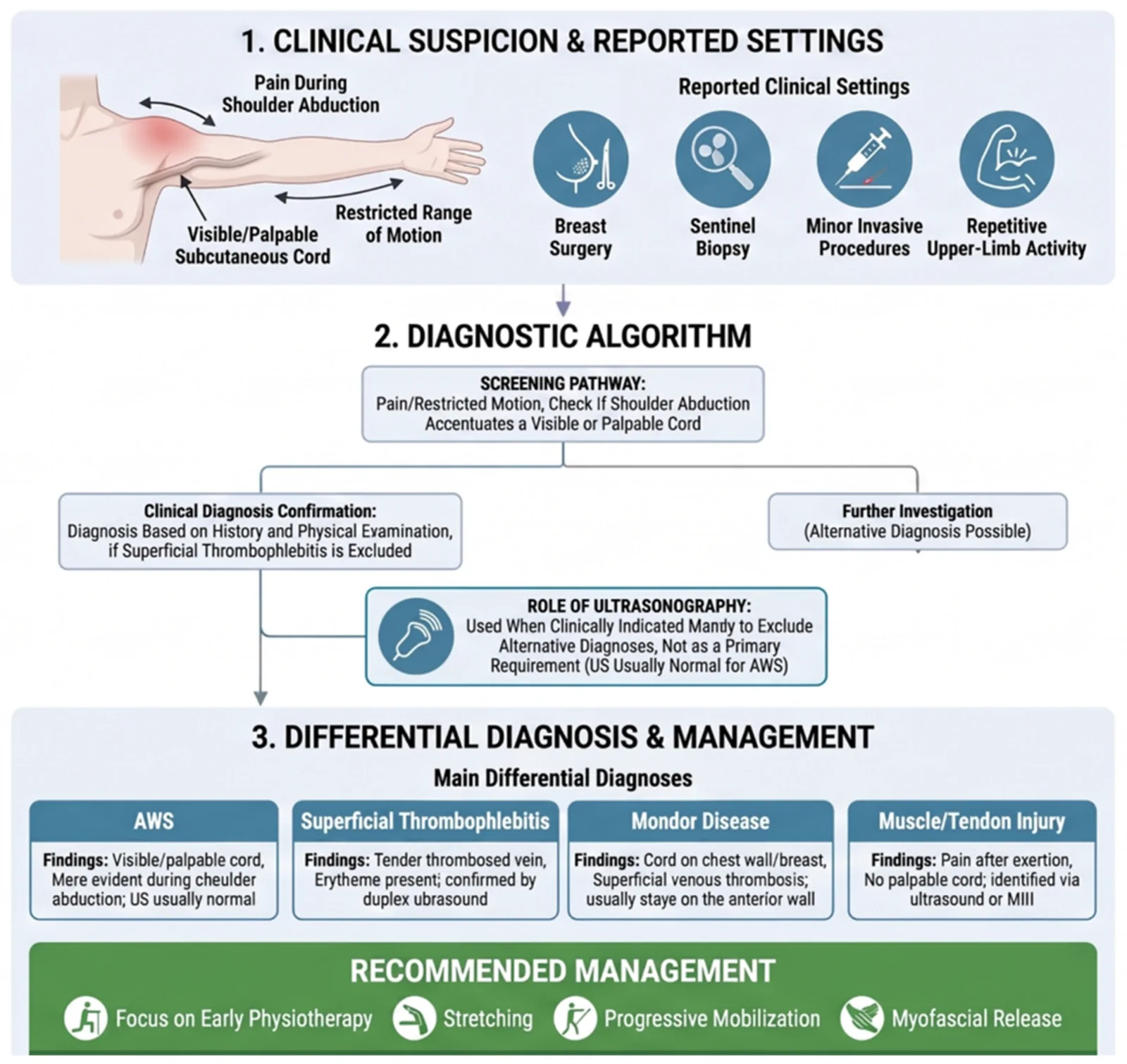
Diagnostic algorithm and clinical management of Axillary Web Syndrome (AWS). Proposed clinical approach for the diagnosis, differential diagnosis, use of ultrasonography, and conservative management of AWS.

The strengths and limitations of the imaging modalities described for AWS are summarized in Table 4, and a proposed diagnostic and management algorithm is presented in Figure 2.

### 4.5. Pathophysiology

Despite more than two decades of investigation, the pathophysiology of Axillary Web Syndrome (AWS) remains incompletely understood. Although the syndrome has traditionally been attributed to injury of superficial lymphatic vessels following axillary surgery, accumulating anatomical, imaging, and histopathological evidence indicates that its biological basis is considerably more complex. Current knowledge suggests that AWS results from a dynamic process involving lymphatic injury, local inflammation, fibrin deposition, tissue remodeling, fibrosis, and, in selected patients, vascular alterations rather than from a single isolated pathological mechanism [10,11,13,15,44].

The earliest hypotheses proposed that the palpable cords represented thrombosed or obstructed lymphatic vessels. Histopathological observations by Reedijk et al. (2006) [10] identified dilated lymphatic channels surrounded by fibrotic tissue, whereas the anatomical study by Leduc et al. (2009) [12] demonstrated that the cords consistently followed the course of superficial lymphatic collectors extending from the axilla toward the upper limb. Together, these investigations provided the first convincing anatomical and pathological evidence supporting a predominantly lymphatic origin of the syndrome.

More recently, however, this concept has evolved considerably. Histopathological findings reported by Johansson et al. [29] demonstrated thrombosis within a D2-40-positive lymphatic vessel undergoing recanalization, providing the first direct evidence that transient lymphatic thrombosis may participate in the early stages of AWS. Nevertheless, because definitive lymphatic thrombosis was demonstrated in only a limited number of specimens, whereas other biopsies revealed nonspecific inflammatory and fibrotic changes, lymphatic thrombosis alone is unlikely to explain the entire clinical spectrum of the disease.

This observation also offers a plausible explanation for one of the characteristic clinical features of AWS: its tendency toward spontaneous resolution. Progressive recanalization of injured lymphatic vessels, accompanied by resolution of inflammation and connective tissue remodeling, may account for the gradual clinical improvement observed in many untreated patients. Although this hypothesis remains speculative, it is biologically consistent with the available histopathological findings and may explain why the syndrome is frequently self-limited.

Additional imaging and pathological investigations have further expanded the understanding of AWS. Furlan et al. (2018) [13] described vascular alterations involving axillary and brachial vessels, whereas lymphoscintigraphic studies by Roman et al. (2022) [14] demonstrated abnormalities in superficial lymphatic drainage, reinforcing the central role of lymphatic dysfunction. More recently, Mizuno et al. (2023) [15] reported vascular thrombosis associated with arterial intimal hyperplasia in a patient with AWS, suggesting that vascular remodeling may also contribute to cord formation in selected individuals. Although these findings remain limited to small observational studies and isolated case reports, they collectively indicate that multiple tissue compartments—including lymphatic vessels, small blood vessels, and surrounding connective tissue—participate in the pathological process.

Based on the currently available evidence, an integrated lymphovascular response to localized tissue injury represents one plausible model for AWS. Surgical dissection, needle puncture, repetitive mechanical stress, sports-related activity, or other minor traumatic events may initiate a localized inflammatory cascade affecting superficial lymphovascular structures. Subsequent fibrin deposition, collagen remodeling, fibrosis, and connective tissue contraction may contribute to the characteristic palpable cords responsible for pain and restricted shoulder mobility [3,35,36,37,38,45]. This integrated model also explains why clinically similar manifestations may arise after markedly different triggering events, as illustrated by both previously published reports and the present case series.

Importantly, none of the currently proposed mechanisms completely explains every clinical presentation of AWS. Rather than representing a single pathological entity, AWS probably reflects a heterogeneous biological response in which the relative contribution of lymphatic injury, thrombosis, inflammation, fibrosis, vascular remodeling, and connective tissue changes varies among individuals. This concept also helps explain the diversity of imaging findings reported across studies and the absence of a single pathological hallmark consistently identified in all patients [25,27,39,40,46].

**Figure 3:**
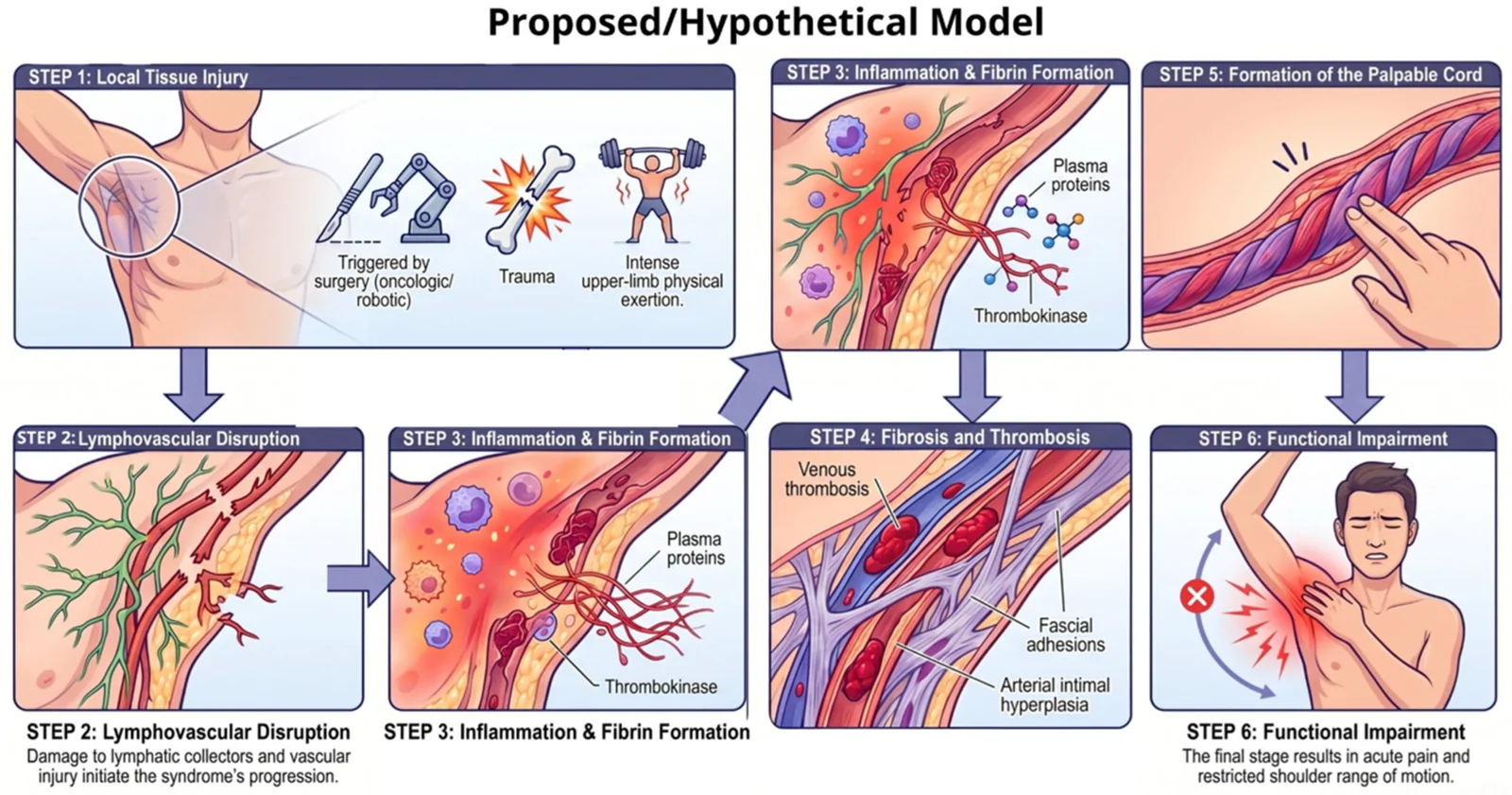
Proposed hypothetical model of the pathophysiological mechanisms potentially involved in Axillary Web Syndrome. The sequence shown represents an integrated conceptual model based on currently available evidence and should not be interpreted as an established biological pathway.

The conceptual pathophysiological model proposed in Figure 3 summarizes the current evidence and integrates the principal mechanisms described in the literature. Rather than representing a definitive sequence of biological events, this model should be interpreted as a practical framework that reflects the present understanding of AWS while highlighting important areas for future anatomical, imaging, and histopathological research.

### 4.6. Treatment and Rehabilitation

The management of Axillary Web Syndrome (AWS) remains predominantly conservative, with the primary objectives of relieving pain, restoring shoulder mobility, improving upper-limb function, and facilitating an early return to daily activities. Although spontaneous resolution has been documented in many patients, untreated AWS may prolong pain, delay postoperative recovery, impair functional performance, and negatively affect quality of life. Consequently, early recognition followed by prompt rehabilitation has become the cornerstone of contemporary management [47,48,49,50].

Current evidence consistently supports physiotherapy as the first-line treatment for symptomatic patients. Therapeutic exercise, particularly progressive stretching and active shoulder mobilization, has demonstrated the most consistent benefits in improving range of motion, reducing pain, decreasing disability, and accelerating functional recovery [48,49,50]. These interventions are believed to improve tissue mobility, reduce mechanical tension within the cords, and promote progressive restoration of upper-limb function.

Manual therapy is frequently incorporated into rehabilitation protocols as an adjunct to therapeutic exercise. Techniques such as myofascial release, scar mobilization, soft-tissue mobilization, and manual lymphatic drainage have shown favorable clinical results, especially when combined with structured exercise programs [5,6,47,48]. However, the isolated contribution of each modality remains uncertain because most published studies have evaluated multimodal rehabilitation protocols rather than individual interventions.

Recent randomized clinical trials further support this multidisciplinary approach. Torres-Lacomba et al. (2022) [6] demonstrated that a physiotherapist-designed rehabilitation program combining manual lymphatic drainage with progressive therapeutic exercises produced greater improvements in pain and shoulder mobility than exercise alone. Likewise, González-Rubiño et al. (2025) [48] reported that a multimodal protocol including stretching, scar massage, and manual tissue release significantly reduced pain, improved shoulder function, and shortened the clinical course of AWS. Collectively, these studies suggest that rehabilitation strategies integrating multiple therapeutic modalities may provide greater clinical benefit than isolated interventions.

Despite these encouraging findings, the current evidence base remains limited. Most available studies are single-center investigations with relatively small sample sizes, heterogeneous rehabilitation protocols, variable outcome measures, and short follow-up periods. Consequently, although conservative rehabilitation is universally accepted as the standard of care, the optimal combination, intensity, frequency, and duration of treatment have not yet been established [47,48,49,50,51,52].

The patients included in the present case series were managed according to these contemporary principles. Conservative treatment, including physiotherapy, progressive stretching, shoulder mobilization, and myofascial release when clinically indicated, resulted in clinical and functional recovery in all three patients within two weeks of follow-up, without the need for invasive intervention. Although conclusions regarding treatment efficacy cannot be drawn from a small case series, the favorable outcomes observed are consistent with the current literature supporting early conservative rehabilitation.

Taken together, the available evidence indicates that successful management of AWS depends less on a single therapeutic technique than on early diagnosis, timely referral for rehabilitation, and individualized treatment according to symptom severity and functional impairment. As recognition of AWS expands beyond the oncologic setting, multidisciplinary rehabilitation strategies should remain the foundation of patient management while higher-quality clinical trials establish standardized treatment protocols.

### 4.7. Clinical Implications

The expanding clinical spectrum of Axillary Web Syndrome has important implications for clinical practice. Although AWS remains most commonly recognized after breast cancer surgery, clinicians should also consider the diagnosis in patients presenting with characteristic axillary cording, pain, and restricted shoulder mobility in non-oncologic settings [23,37]. This is particularly relevant across vascular surgery, rehabilitation medicine, sports medicine, orthopedics, dermatology, radiology, and primary care, where limited familiarity with AWS may contribute to delayed recognition and unnecessary investigations. Careful clinical examination remains central to diagnosis, while ultrasonography is most useful when needed to exclude competing vascular or structural conditions [20,22,23,28].

From a broader perspective, recognition of AWS across different clinical settings highlights the need for standardized diagnostic criteria and further investigation of whether postoperative and non-oncologic presentations represent distinct phenotypes or different clinical expressions of a shared lymphovascular process [29,37]. Improved awareness and interdisciplinary collaboration may facilitate earlier diagnosis and more consistent investigation of this underrecognized syndrome, while appropriate conservative rehabilitation remains the mainstay of management [24,25].

### 4.8. Strengths and Limitations

The present study integrates illustrative clinical cases with a contemporary synthesis of the available evidence regarding the epidemiology, pathophysiology, diagnosis, imaging assessment, differential diagnosis, and management of AWS. A particular strength and novel aspect of the study is the inclusion of both oncologic and non-oncologic presentations within the same clinical framework. The two non-oncologic cases, temporally associated with intense or repetitive upper-limb activity, add to the limited literature describing AWS outside the traditional breast cancer setting [23,35]. Importantly, these observations should not be interpreted as evidence of a causal relationship between mechanical activity and AWS but rather as clinically relevant presentations that broaden the contexts in which the syndrome may be recognized.

Several limitations should be acknowledged. First, the retrospective and descriptive nature of the case series and the small number of patients preclude conclusions regarding incidence, risk factors, prognosis, causality, or treatment effectiveness. Second, histopathological confirmation was not available, and diagnosis was established clinically after exclusion of relevant alternative conditions, reflecting routine clinical practice. Third, standardized pain scores, goniometric measurements of shoulder range of motion, and validated functional assessment instruments were not systematically collected; therefore, the reported clinical and functional recovery within two weeks reflects clinical follow-up rather than quantitatively measured treatment outcomes. Finally, the accompanying literature review was narrative rather than systematic and is therefore subject to potential selection bias.

Despite these limitations, the integration of the three clinical cases with contemporary evidence provides a clinically oriented perspective on AWS and highlights areas requiring further investigation, particularly its non-oncologic presentations, underlying mechanisms, diagnostic standardization, and rehabilitation outcomes [23,29,37].

### 4.9. Future Perspectives

Although substantial progress has been made in understanding Axillary Web Syndrome over the past two decades, several important questions remain unanswered. Future prospective multicenter studies should establish standardized diagnostic criteria, allowing for more reliable estimates of disease incidence and facilitating comparisons among epidemiological studies. Such standardization is particularly important if AWS is to be recognized as a clinical syndrome extending beyond the postoperative breast cancer setting.

Further anatomical, histopathological, and advanced imaging investigations are also needed to clarify the relative contribution of lymphatic injury, vascular remodeling, inflammation, fibrosis, and connective tissue changes to cord formation. Improved understanding of these biological mechanisms may help explain the clinical heterogeneity observed among patients and determine whether postoperative and non-oncologic presentations represent distinct phenotypes or different manifestations of the same pathological process.

From a therapeutic perspective, adequately powered randomized clinical trials comparing standardized rehabilitation protocols remain a priority. Future studies should evaluate not only treatment efficacy but also the optimal timing, intensity, duration, and combination of rehabilitation strategies, together with long-term functional outcomes and patient-reported quality-of-life measures.

Finally, increasing recognition of AWS in non-oncologic settings opens new opportunities for interdisciplinary research involving vascular surgery, rehabilitation medicine, sports medicine, radiology, orthopedics, dermatology, and primary care. Expanding collaboration across these specialties will be essential to improve early diagnosis, refine disease classification, optimize management strategies, and ultimately provide a more comprehensive understanding of this frequently underrecognized clinical syndrome.

## 5. Conclusions

Axillary Web Syndrome may occur in both oncologic and non-oncologic settings and should be considered in patients presenting with characteristic axillary cording, pain, and restricted shoulder mobility. Diagnosis remains primarily clinical, with ultrasonography serving as a complementary tool to exclude competing conditions. Conservative rehabilitation is the mainstay of management. Further prospective studies are needed to clarify the epidemiology, pathophysiology, diagnostic criteria, and clinical course of AWS across different patient populations.

## Figures and Tables

**Table 1:** Clinical characteristics of the three patients with Axillary Web Syndrome.

Variable	Case 1	Case 2	Case 3
Sex	Male	Female	Female
Age	34	35	41
Clinical setting	Non-oncologic	Breast cancer surgery	Non-oncologic
Trigger	Squash tournament	Mastectomy	Strenuous workday involving prolonged repetitive upper-limb activity (commercial kitchen)
Previous breast surgery	No	Yes	No
Previous trauma	No	No	No
Palpable axillary cord	Yes	Yes	Yes
Visible cord	Yes	Not described	Yes
Shoulder pain	Yes	Yes	Mild
Restricted shoulder movement	Yes	Yes	Yes
Ultrasonography	No evidence of superficial thrombophlebitis or structural abnormalities	No evidence of superficial thrombophlebitis or structural abnormalities	No evidence of superficial thrombophlebitis or structural abnormalities
Initial differential diagnoses	Superficial thrombophlebitis, muscle strain, lymphangitis, soft tissue injury	Superficial thrombophlebitis, postoperative fibrosis, lymphatic complications, local inflammatory conditions	Superficial thrombophlebitis, muscle injury, lymphangitis, axillary lymphadenopathy
Treatment	Physiotherapy, stretching, myofascial release, shoulder mobilization	Manual therapy, myofascial release, stretching, shoulder mobilization	Physiotherapy, stretching, shoulder mobilization
Outcome	Complete clinical and functional recovery within 2 weeks	Clinical and functional recovery within 2 weeks	Complete clinical and functional recovery within 2 weeks

**Table 2:** Selected reports illustrating the clinical spectrum of Axillary Web Syndrome.

Author (Year)	Clinical Setting	Proposed Trigger	Management	Outcome
Schuitevoerder et al. (2016) [16]	Melanoma	Sentinel lymph node biopsy	Conservative rehabilitation	Clinical improvement
Wei et al. (2013) [31]	Breast surgery	Secondary breast-conserving surgery	Conservative treatment	Clinical improvement
Kitajima et al. (2023) [17]	Non-oncologic	Venous blood sampling	Conservative treatment	Complete recovery
Rahmani et al. (2025) [18]	Non-oncologic	Punch biopsy	Conservative treatment	Clinical improvement
Denton & Shehab (2025) [19]	Pediatric patient	No previous surgery or malignancy	Physiotherapy	Clinical improvement
Present study (2026)	Non-oncologic	Squash-related repetitive upper-limb activity; occupational repetitive upper-limb activity	Physiotherapy, stretching, shoulder mobilization	Complete functional recovery

**Table 3:** Differential diagnosis of Axillary Web Syndrome.

Condition	Clinical Features	Ultrasonographic Findings	Distinguishing Features
Axillary Web Syndrome	Palpable or visible cord extending from the axilla, pain during shoulder abduction, restricted shoulder mobility	Usually normal or nonspecific; no superficial venous thrombosis	Cord becomes more evident with shoulder abduction; diagnosis primarily clinical
Superficial thrombophlebitis	Painful indurated cord following the course of a superficial vein; erythema and local tenderness may be present	Thrombosed superficial vein with absent or reduced compressibility ± intraluminal thrombus	Venous involvement confirmed by duplex ultrasonography
Mondor’s disease	Tender subcutaneous cord involving the anterolateral chest wall or breast	Thrombosis of superficial thoracoepigastric or lateral thoracic veins	Usually affects the chest wall rather than the axilla or medial arm
Lymphangitis	Painful linear erythema associated with fever or systemic symptoms	Usually nonspecific; no cord-like vascular lesion	Infectious presentation with inflammatory skin changes
Muscle or tendon injury	Localized pain after physical activity, aggravated by movement	Muscle or tendon abnormalities when present	Absence of palpable cord
Postoperative fibrosis/scar contracture	Localized stiffness and restricted mobility after surgery	Fibrotic tissue without cord-like structure	Limited to the surgical field
Adhesive capsulitis	Progressive shoulder pain and global restriction of active and passive motion	Normal shoulder soft tissues or secondary findings	No palpable axillary cord
Axillary lymphadenopathy	Palpable nodules or masses in the axilla	Enlarged lymph nodes with preserved or altered morphology	Nodular rather than linear lesion

**Table 4:** Imaging modalities used in the evaluation of Axillary Web Syndrome, their typical findings, clinical applications, and limitations.

Imaging Modality	Typical Findings	Clinical Role	Main Limitations
Ultrasonography (US)	Usually normal or nonspecific; occasionally demonstrates thin cord-like structures within the superficial subcutaneous tissue	First-line imaging to exclude superficial thrombophlebitis and other structural abnormalities	Normal findings do not exclude AWS; low sensitivity for direct visualization of cords
Duplex Doppler ultrasonography	Normal superficial veins without thrombosis or flow abnormalities	Differentiates AWS from superficial thrombophlebitis and other venous disorders	Does not confirm the diagnosis of AWS
Lymphoscintigraphy	Abnormal superficial lymphatic drainage and delayed lymphatic transport	Demonstrates lymphatic dysfunction; mainly used in research	Limited availability and not recommended for routine diagnosis
Histopathological examination	Lymphatic vessel wall thickening, fibrosis, and occasional lymphatic thrombosis with recanalization	Improves understanding of disease mechanisms	Invasive; restricted to research settings and not indicated for routine diagnosis

## Data Availability

The data presented in this study are available from the corresponding author upon reasonable request. The data are not publicly available because they contain information that could compromise the privacy of the study participants.
